# Evaluating rates of chiropractic use and utilization by patient sex within the United States Veterans Health Administration: a serial cross-sectional analysis

**DOI:** 10.1186/s12998-023-00497-x

**Published:** 2023-08-10

**Authors:** Sarah E. Graham, Brian C. Coleman, Xiwen Zhao, Anthony J. Lisi

**Affiliations:** 1 VA Connecticut Healthcare System, West Haven, CT USA; 2 Yale School of Medicine, New Haven, CT USA; 3 Yale Center for Analytical Sciences, New Haven, CT USA

**Keywords:** Veterans health services, Chiropractic, Facilities and services utilization, Usage, Sex distribution

## Abstract

**Background:**

Within the United States Veterans Health Administration (VHA), the number of patients using healthcare services has increased over the past several decades. Females make up a small proportion of overall patients within the VHA; however, this proportion is growing rapidly. Previous studies have described rates of VHA chiropractic use; however, no prior study assessed differences in use or utilization rates between male and female veterans. The purpose of this study was to assess rates of use and utilization of chiropractic care by sex among VHA patients receiving care at VHA facilities with on-station chiropractic clinics.

**Methods:**

A serial cross-sectional analysis of VHA national electronic health record data was conducted in Fall 2021 for fiscal year (FY) 2005–2021. The cohort population was defined as VHA facilities with on-station chiropractic clinics, and facilities were admitted to the cohort after the first FY with a minimum of 500 on-station chiropractic visits. Variables extracted included counts of unique users of any VHA on-station facility outpatient services, unique users of VHA on-station facility chiropractic services, number of chiropractic visits, and sex. To calculate use, we determined the proportion of patients of each sex who received chiropractic services to the total patients of the same sex receiving any outpatient care within each facility. To calculate utilization, we determined the number of chiropractic care visits per patient per fiscal year. A linear mixed effects model was applied to examine the difference in chiropractic care utilization by sex.

**Results:**

The percentage of female VHA on-station chiropractic patients increased from 11.7 to 17.7% from FY2005–FY2021. Among VHA facilities with on-station chiropractic care, the percentage of female VHA healthcare users who used chiropractic care (mean = 2.3%) was greater than the percentage of male VHA healthcare users who used chiropractic care (mean = 1.1%). Rates of chiropractic utilization by sex among VHA facilities with on-station chiropractic clinics were slightly higher for females (median = 4.3 visits per year, mean = 4.9) compared to males (median = 4.1 visits per year, mean = 4.6).

**Conclusion:**

We report higher use and utilization of VHA chiropractic care by females compared with males, yet for both sexes rates were lower than in the private US healthcare system. This highlights the need for further assessment of the determinants and outcomes of VHA chiropractic care.

## Background

The Veterans Health Administration (VHA) is the largest integrated health care system in the United States, providing care at 1298 health care facilities, including 171 VHA Medical Centers and 1113 outpatient sites of care [1]. In 2021, over 9 million veterans were enrolled in VHA health care programs, and 6.8 million of these veterans received 78.8 million VHA visits [1–3].

In 2021, there were over 2 million female veterans (10.7% of all veterans), and 550,000 of those received VHA health care [2, 3]. Female veterans are the fastest growing demographic group utilizing VHA health care with a nearly three-fold increase from fiscal year (FY) 2000–2015 [4]. The VHA projects that by 2045 female veterans will make up one in five of all US veterans [5].

The prevalence of musculoskeletal disorders for female veteran patients is high. Among female VHA patients, those diagnosed with a musculoskeletal condition increased six-fold from FY 2000–2015 [4]. Multiple studies comparing female to male veteran populations indicate that female veterans experience a greater number of pain sites and exhibit higher prevalence of musculoskeletal conditions [4, 6–8]. Moreover, a significant proportion of female veterans who regularly attend appointments at VHA medical centers utilize these services to manage persistent pain issues, particularly low back pain, and musculoskeletal pain is the primary reason for seeking treatment at VHA [9, 10].

A growing option for treating musculoskeletal conditions in VHA is chiropractic care. Since 2004, when VHA began providing on-station chiropractic services (that is, care delivered in VHA facilities by VHA chiropractors) at a limited number of facilities, the uptake of this new service has been expanding [11]. In this period, the proportion and number of veterans using on-station chiropractic services significantly increased, with rates of chiropractic visits increasing an average of 15% year-over-year [12, 13]. Since 2004, over 280,000 unique patients have received chiropractic care on-station at VHA facilities [14].

Currently, there is limited data regarding sex differences in use of chiropractic within the VHA despite the increasing use of this service. There have been no reports of the rates of VHA chiropractic care use among VHA healthcare users by sex, whereas estimated rates of use by sex for the entire US population range from 5.7 to 11.1% for females and 4.6 to 9.4% for males [15, 16]. Analyses of the patient population seen in VHA chiropractic clinics have reported 15.8% to 17.1% are female [13, 17]. While a lower percentage can be expected due to the overall sex distribution within VHA, this is in contrast with the population of US chiropractic private practice patients, of which approximately 60% are female [18–20].

There is a paucity of research regarding utilization—the number of visits per patient per time period—for VHA chiropractic patients. Reports within the veteran population indicate a range of on-station chiropractic visits per veteran, varying from a single visit to upwards of 73, with an annual per patient average of approximately 6 visits [21, 22]. Similarly, within the general US population there is wide variation in estimates of average annual chiropractic visits per patient. One study reported a median number of 3.5 chiropractic visits per person annually, while other studies reported average visits per patient per year range from 6.7 to 9.8, and monthly visit counts ranging from 0 to 14 [23–26]. One study reported differences in utilization rates of chiropractic care by sex, finding the average number of annual visits per patient was 11.5 for females and 9.4 for males [27].

As the number of female veterans receiving VHA healthcare continues to increase and because the prevalence of musculoskeletal diagnoses for this population is high, efforts are needed to optimize delivery of evidence-based care, including non-pharmacological approaches, for this population [4, 6–8]. The current limited knowledge of VHA chiropractic use and utilization rates by sex presents an obstacle to modeling and assessing appropriate and equitable access and quality. Accordingly, we conducted a program assessment project to measure and compare national rates of use (as the proportion of patients receiving chiropractic services at a facility) and utilization (as the number of chiropractic care visits by patient) of on-station VHA chiropractic care, stratified by patient sex. The aim of this study was to assess rates of use and utilization of on-station chiropractic care, by sex, among VHA patients receiving care at VHA facilities with on-station chiropractic clinics.

## Methods

This project was a serial cross-sectional analysis of VHA national electronic health record data from FY 2005 to FY 2021 (October 1, 2004–September 30, 2021). This study period represented the first 17 FYs where chiropractic care was available to veterans and where complete FY data were accessible at the time of this study.

All data were obtained from VHA’s Corporate Data Warehouse. To measure use at facilities in which the patient population had the opportunity to receive on-station chiropractic services, we limited our sample to a cohort of facilities providing a threshold amount of such services. A facility entered the cohort in the first FY it provided a minimum of 500 on-station chiropractic visits. We operationalized this 500-visit threshold to allow for new clinics to become established within a facility. By limiting our sample of facilities to only those providing a threshold of on-station chiropractic visits, we aimed to assess a population of patients that had the potential to receive such care in established clinics. We hypothesized that using a minimum threshold of patient visits to define established clinics would provide a more accurate denominator to estimate use and utilization by excluding facilities. Once a facility entered the cohort, it remained for the duration of the study timeframe.

From this cohort we extracted the number of total facility unique patients receiving any outpatient services during each FY. We obtained the number of unique patients receiving any chiropractic visits, in total and stratified by sex, by identifying chiropractic care visits using an administrative data identifier ("Stop Code 436—Chiropractic Care") for each fiscal year.

This was a program assessment project, and the VA Connecticut Healthcare System’s Research Department determined the work did not require Institutional Review Board review.

### Statistical analysis

We summarized demographic and facility characteristics by fiscal year using descriptive statistics. The association between sex and utilization of chiropractic service was examined using linear mixed effects models. The dependent variables in the models were the chiropractic service use rate and chiropractic service utilization.

The chiropractic service use rate was defined as the proportion of chiropractic care patients among all patients receiving outpatient care at a given facility. Chiropractic service utilization referred to the number of visits per chiropractic care patient of the same sex in each facility. To account for potential correlation within the same facility, the variable “Facility” was included as the random effect in the model, thus addressing possible clustering effects.

In analyzing the trend in chiropractic care utilization, time was treated as a continuous variable centered at 2005. The analysis assumed a linear relationship between the dependent variable and independent variable (time). An interaction term of time and sex was included to study if sex modified the growing trend of chiropractic care usage. All analyses were conducted in R Statistical Software (v4.2.2) [28].

## Results

The cohort of facilities and patients by fiscal year is presented in Table 1. Over the study timeframe of FY2005 to FY2021, the number of facilities meeting the threshold for providing on-station chiropractic care increased from 15 to 171, and the total number of unique patients seen at all eligible facilities during an individual FY increased from 557,284 (F% = 5.1) to 6,690,841 (F% = 9.9). Unique patients receiving on-station VHA chiropractic care by FY increased from 3475 (F% = 11.7) to 81,706 (F% = 17.7).

**Table 1:** Total and chiropractic patients at VHA facilities with minimum 500 on-station chiropractic visits per FY

Fiscal year	VHA facilities	Total patients (% female)	Chiropractic patients (% female)	% of Populations using chiropractic care		
				Total	Male	Female
2005	15	557,284 (5.1)	3475 (11.7)	0.6	0.6	1.4
2006	26	1,071,500 (5.6)	8138 (13.2)	0.8	0.7	1.8
2007	33	1,257,093 (5.8)	11,259 (13.7)	0.9	0.8	2.1
2008	34	1,392,141 (6.2)	14,094 (14.0)	1.0	0.9	2.3
2009	34	1,435,291 (6.5)	16,791 (14.1)	1.2	1.1	2.6
2010	37	1,584,473 (6.6)	18,490 (13.6)	1.2	1.1	2.4
2011	43	1,841,167 (6.8)	21,318 (13.9)	1.2	1.1	2.4
2012	44	1,895,504 (7.0)	24,053 (12.3)	1.3	1.2	2.6
2013	46	2,035,407 (7.3)	26,080 (15.0)	1.3	1.2	2.6
2014	51	2,391,258 (7.5)	30,116 (15.5)	1.3	1.1	2.6
2015	59	2,748,822 (7.7)	37,436 (15.3)	1.4	1.3	2.7
2016	71	3,341,124 (8.1)	44,117 (15.9)	1.3	1.2	2.6
2017	77	3,573,834 (8.4)	47,489 (16.2)	1.3	1.2	2.6
2018	90	4,037,229 (8.7)	51,199 (16.1)	1.3	1.2	2.3
2019	119	4,750,436 (9.0)	66,344 (16.4)	1.4	1.3	2.5
2020	144	5,509,271 (9.6)	66,535 (17.0)	1.2	1.1	2.1
2021	171	6,690,841 (9.9)	81,706 (17.7)	1.2	1.1	2.2

In each reported year, the percentage of female patients was higher for chiropractic care compared to overall VHA healthcare users. The average annual growth rate of on-station chiropractic users was 22.5%, while the average annual growth rate stratified by sex was 34.6% for females and 20.9% for males.

### Use rates

**Fig. 1:**
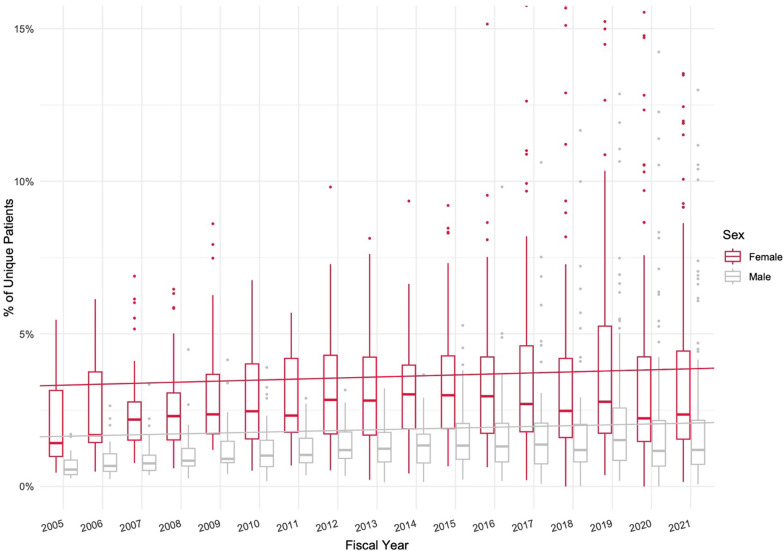
VHA chiropractic care use rates, by sex, by FY

Use rates are presented in Fig. 1. Throughout the course of this study the percentage of all female VHA patients using chiropractic care increased from 1.4 to 2.4%, a year-by-year increase of 0.03% (*p* < 0.001) after adjusting for sex and the interaction between sex and time. The percentage of all male VHA patients using chiropractic care increased from 0.6 to 1.1%, a year-by-year increase of 0.03% (*p* = 0.006). In each FY, the median percentage of the facility female population using chiropractic care was greater than the median percentage of the facility male population. The difference in the trend of increasing use between male and female patients was not statistically significant.

### Utilization rates

**Fig. 2:**
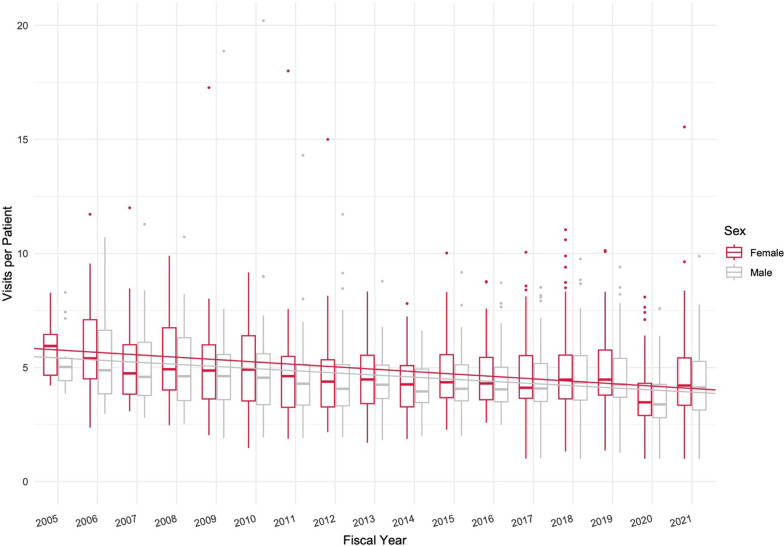
VHA chiropractic care utilization rates, by sex, by FY

Utilization rates are presented in Fig. 2. Within our cohort, the average number of visits per patient was 4.7 visits per year (SD = 1.8). Overall, rates of utilization declined slightly over the course of the study by an average of 0.1 visits per year (*p* < 0.001) for both sexes. Throughout this study, the average female utilization rate was 4.9 visits per year (SD = 1.9, median = 4.3, IQR = 3.5, 5.5) and the average male utilization rate was 4.6 visits per year (SD = 1.7, median = 4.1, IQR = 3.4, 5.2). Overall, there was a slight decrease in utilization rates for both sexes over time, with no statistically significant difference in the rates of change year-over-year. A statistically significant difference was found in utilization rates stratified by sex, with female patients receiving a median of 0.4 more visits per year (*p* = 0.01) than male patients.

## Discussion

To our knowledge, this is the first report examining national use and utilization rates of on-station VHA chiropractic care by patient sex. By limiting our sample of facilities to only those providing a threshold of on-station chiropractic visits, we aimed to assess a population of patients that had the potential to receive such care in established clinics. From FY2005 to FY2021, the number of VHA facilities meeting our minimum amount of on-station chiropractic care increased substantially. This is consistent with prior work assessing the ongoing expansion of VHA chiropractic clinics nationally [12, 13]. This increase may be related to the natural diffusion of change across the VHA system and in response to legislation passed in 2018 which mandated expansion of on-station chiropractic clinics [12, 29].

During our study timeframe, the overall female patient population in this group of facilities increased from 5.1 to 9.9%. This finding is in line with demographic changes within the VHA and increasing VHA healthcare usage rates for female veterans [2, 3]. One prior publication reported female veterans are the fastest growing demographic group using VHA healthcare—with use nearly tripling during their observation period [4].

In our cohort of facilities, the average female use rate of chiropractic care was higher than the male usage rate year-over-year. Rates of use for both sexes increased throughout the study period; however, there was no statistically significant difference in the trend of year-on-year increasing use rates by sex. Despite higher rates of use among the female VHA population, most chiropractic patients seen at on-station VHA chiropractic clinics were male, which is expected considering that females make up only 10.7% of the overall VHA population [3]. This expectedly contrasts with multiple studies reporting female patients make up well over 50% of chiropractic patient populations in non-VHA clinical settings [18–20].

We found utilization of VHA chiropractic care was slightly higher for females compared to males. Within our cohort, on average females received 4.9 visits per year and males 4.6 visits and median rates of utilization were 4.3 for females and 4.1 for males, respectively. This is approximately one annual visit higher on average than a previous VHA study, but similarly showed a pattern of higher overall utilization rates among female than male patients [21]. This same study also found that, when stratified by the number of visits, there were more female veterans in the 7+ visit quartile than any of the other three quartiles [21]. In comparison, reported chiropractic utilization rates among the general US population vary greatly, with median utilization reported as low as 3.45 visits per year (range: 1 to 56 visits per year) while other studies reported averages of approximately 6.7 visits per year [23–25, 27].

Although use of VHA chiropractic care is growing, in the last year of our study (FY 2021) only 1.2% of our cohort population used this service. While assessing the overall rate of chiropractic use in VHA was beyond the scope of our project, this result is consistent with prior work demonstrating a 1.5% overall VHA annual chiropractic use rate, and comparatively much lower than the general US population where rates of 10.3% to 13.7% have been reported [12, 15, 18]. Non-VHA studies commonly report higher overall rates of use for both sexes compared to VHA, with female patients using more chiropractic care than males [15, 16, 19, 20, 30]. The estimated rates of use by sex for the entire US population range from 5.7 to 11.1% for females and 4.6 to 9.4% for males, respectively [15, 16]. This is in line with previous studies indicating females use healthcare with greater frequency than males and that the prevalence of musculoskeletal pain is higher among the female population [16, 31].

As the patient population using VHA healthcare continues to change, there is a need to better the understand characteristics of VHA chiropractic patients, especially for populations with a high incidence rate of musculoskeletal conditions such as female veterans [4, 7, 10]. The health services implications of this research suggest use of chiropractic care by females will continue to increase, highlighting the need for clinicians to be competent in women’s health considerations in case management. Additionally, VHA’s implementation of chiropractic care has not reached full market penetration, thus at present veterans may be facing barriers to accessing VHA chiropractic care [11].

### Limitations

Our results are limited to care provided on-station at VHA facilities and do not account for patients receiving VHA purchased care in the community, which has been shown to be growing [12]. Although we believe our method of limiting facilities to those with a minimum threshold of 500 chiropractic visits yields an appropriate denominator for our study questions, this approach is untested. We also considered use and utilization by fiscal year, which does not account for patient care spanning across the transition between fiscal years. Thus, we may potentially be undercounting service utilization across an episode of care in these cases. We did not attempt to account for differences associated with various chiropractic clinic staffing levels, nor for clinic capacity changes related to the timing of the COVID-19 pandemic, which may have affected chiropractic service availability, use, and utilization. Finally, our assessment was limited to patient sex as it is recorded in the VHA electronic health record. During the timeframe of this study, patient demographic data did not include gender identity. As of January 2022, VHA has begun collecting self-identified gender identity data across enrollment, administrative, and health records systems as part of Directive 1341 [32].

### Future studies

Additional work is needed to assess the clinical, socio-demographic, or systems factors, such as policy environment, temporal patterns across facilities, and other facility level factors, potentially impacting male or female patients’ use of VHA chiropractic care on-station. Future observational database studies and qualitative work assessing stakeholder perceptions may help identify facilitators and barriers to accessing and using VHA chiropractic care. Such studies should also strive to explore the impact of gender identity in this context.

## Conclusions

We report higher use and utilization of on-station VHA chiropractic services for female patients compared with their male counterparts, yet overall both are lower than the general US population. Further research is needed to assess the impact of patient sex on the use and utilization of VHA chiropractic care.

## Data Availability

The datasets generated and/or analyzed during the current study are not publicly available due to VHA privacy and information security policies, but de-identified datasets are available from the corresponding author on reasonable request.

## Abbreviations

VHA: United States Veterans Health Administration
FY: Fiscal year
